# Household Hints and Recipes

**Published:** 1883-04

**Authors:** 


### Household Hints & Recipes.

GLYCERIN FOR THE HAIR.—

Balsam of Peru, 5,000 parts.
Glycerin, 44,000 parts,
Spirits of Wine, 25,000 parts.
Essence of violets, 12 parts.

---

TO REMOVE FRECKLES.—Dr. J. V. Shoemaker, of Philadelphia, Pa., states that the careful application of a small piece of the ointment of the oleate of copper at night upon retiring will usually remove freckles. The oleate of copper ointment should be prepared by dissolving one drachm of the salt of oleat of copper in sufficient oleo-palmitic acid to make a soft ointment.

---

ONION-PEEL FOR DYEING ORANGE YELLOW.—The simple decoction of onion peel is said to produce upon glove leather an orange yellow superior in lustre to any other. It is also said to be suitable for mixing with light bark shades, especially willow bark, and as a yellow for modulating browns. The onion dye is said to fix itself readily, even upon leathers which resist colors, and colors them well and even.

---

FOR ITCHING PILES.—Dr. J. F. Gibbon (San Francisco) recommends the following:

℞
Hyd. chlor. cor., 8 grains.
Aquæ, 8 ounces.
Spirit. lavand. compound, 6 drachms.

Apply three times a day.

Dr. A. V. Banes (St. Joseph, Mo.) prescribes as an application:

℞

Goulard's create, ½ ounce.

Ung. hydrarg. nit., ½ ounce.

Adipis or vaseline, ½ ounce.

Morph. acetat., 1 scruple.

—*Medical Brief.*

---

TO CLEAN PAINT.—When painted work is badly discolored, put a tablespoonful of ammonia water into a quart of moderately hot water, and with the aid of flannel, wipe off the surface. Rubbing is not necessary. When the discoloration is not great, the following method is preferable: With a piece of clean flannel wet with clean warm water, and then squeezed nearly dry, take up as much whiting, of the best quality, as will adhere, apply this with moderate rubbing to the painted work, and afterwards wash the surface with clean water, and rub it dry with chamois leather. This method is superior to the use of soap, requires but half the time and labor, and leaves the surface cleaned, looking as good as new. It will not injure delicate colors.

---

FELONS.—Dr. G. W. Baylor writes as follows to the *Medical Brief:*

Take olive oil, water of ammonia, hydrochloric acid; of each, 2 ounces.

Mix, first, the oil and ammonia, and lastly add the acid gradually, keep it corked tightly, shake thoroughly, and apply to felon three or four times daily.

This, in my opinion, seldom fails to effect a cure in from two to four days. Later in the disease, when the pus has formed, use the lancet.

---

GOLDEN CERATE FOR CORNS.—

Yellow wax, 5 ounces.
Sulphate of zinc, 678 grains.
Oxide copper, 220 grains.
Verdigris, 220 grains.
Borax, 220 grains.
Red chalk, 678 grains.

After a long, fatiguing walk, the feet, especially the heels, are affected by a little white blister full of serosity, looking like a bulb produced by a burn. It is a passing inconvenience. Prick it carefully, and let the water out without breaking the skin; apply a little linen cloth with cold cream, and refrain from long walks. This is simple, and sufficient to cure it.—*Perfumer and Milliner.*

---

INCOMBUSTIBLE CURTAINS.—The following is given (*Cassell's Magazine*) as M. Martin's preparation to render curtains and similar fabrics incombustible:

Sulphate of ammonia (pure), 80 parts.
Carbonate of ammonia, 25 parts.
Boracic acid, 30 parts.
Borax (pure), 12 parts.
Starch, 20 parts.
Water (pure or distilled), 1,000 parts.

The fabrics are saturated with the above solution while hot, and then dried and ironed in the usual manner.

COLD CREAM.—Cold cream may be made in two different ways: either as a mechanical mixture, like any other ointment, or as an emulsion proper, as it always should be made. Any practical pharmacist will understand that the proportions of ingredients which give an unobjectionable *ointment* must give a poor and thin *emulsion*. The U. S. P. formula, for instance: if the mixture be "whipped," as it ought to be, it will become much too soft, and can then easily be poured out of the jar. Another thing, which is generally overlooked, is that cold cream is not one of those preparations where the attention can be divided between it and other duties—stirring it, so to speak, between hands. If stirred till nearly or quite cold, and left to itself, it *sets*; if it be now stirred again, it will soon separate. If, on the contrary, it be whipped up for some time (instead of allowing it to set), it will continue permanently and uniformly soft.

---

BLACK INK FOR RUBBER STAMPS.—

Aniline black, ½ ounce.
Pure alcohol,
Concentrated glycerin, of each, 15 oz.

Dissolve the aniline black in the alcohol and add the glycerin.

---

REMEDY FOR SWEATING HANDS OR FEET.—A writer in the *British Medical Journal* recommends for this the painting of the surface affected, with the tincture of belladona. An ointment of the same could also be offered with advantage in certain cases. In case of the hands, a "bracelet" painting of the tincture about the wrist has proven successful.

---

ADHESIVE LIQUID FOR METAL.—Soften half an ounce of common glue, add about a quarter of an ounce of coarse sugar or candy, and half an ounce of gum-arabic; boil and stir carefully. Useful for applying labels to tin. When reduced to dryness, forms a good "marine glue."—*Monthly Magazine of Pharm.*

---

GLUE TO RESIST FIRE.—Mix a handful of quicklime with four ounces of linseed oil, boil to a good thickness, then spread on some plates in the shade, when it will become exceedingly hard, but may be easily dissolved over the fire, and used as ordinary glue.—*Monthly Magazine of Pharm.*

HARNESS SOAP.—Take resin soap, 2 lbs.; sperm oil, ¾ lb. Digest the soap with a quantity of boiling water just sufficient to thoroughly soften it, when it may be triturated with the warm oil and a sufficient quantity of fine boneblack until a uniform paste is obtained. Ordinary unmixed soap turns brown many of the black pigments in use. The addition of oil is a great improvement.

---

COMPOSITION FOR REMOVING PAINT.—Mr. H. P. Heyhoe, of Stowmarket, has recently patented a preparation which will soften paint, varnish, or japan, so that they can be easily removed from old surfaces, and which can also be used for removing oxide or dirt from the surface of metals, and, when diluted, for cleaning paint, etc. It is a solution of caustic soda, about twice the strength of that in the Pharmacopœia, made from quicklime and common carbonate of soda, and containing, in about 140 gallons, 30 lbs. to 35 lbs. of treacle and 10 stones of flour. A gallon of carbolic acid has to be added to this quantity to prevent decomposition. It is difficult to see the advantages this mixture has over a solution of caustic soda of the same strength.

---

TO DESTROY MOTHS.—The *American Agriculturist* states that benzine will utterly destroy moths, eggs, and larvæ. If furniture, furs, flannels, wool, etc., containing moths, be thoroughly saturated with benzine once, this will be sufficient, and the odor of the benzine will pass off in a few days. The foregoing formulas are copied from the *Chemist and Druggist.*

---

OIL OF EGGS: A VALUABLE RECIPE.—A German apothecary's apprentice describes the mode of preparing "oil of eggs" as follows: "I call on the lady of the house for one dozen of eggs; I boil the eggs, separate the yolks from the whites; the clerks eat the yolks, the white is for the apprentice; into the bottle I pour oil of poppy seed."

---

BLEACHING VEGETABLE TISSUES.—It is stated that a cold five to six per cent. solution of borax will remove all the red, blue, purple, or violet coloring matter from vegetable cells, leaving the green chlorophyll intact.—*Amer. Quarterly Microscop. Jour.*

